# Time motion study using mixed methods to assess service delivery by frontline health workers from South India: methods

**DOI:** 10.1186/s12960-018-0279-7

**Published:** 2018-04-02

**Authors:** Samiksha Singh, Sanjeev Upadhyaya, Pradeep Deshmukh, Amol Dongre, Neha Dwivedi, Deepak Dey, Vijay Kumar

**Affiliations:** 1 Indian Institute of Public Health-Hyderabad, Public Health Foundation of India, Plot #1, Amar co-op society, Kavuri Hills, Madhapur, Hyderabad, 500033 India; 2 UNICEF Hyderabad Field Office, Hyderabad, India; 3 Mahatma Gandhi Institute of Medical Sciences, Sevagram, Wardha, India; 4 Department of Community Medicine, Sri Manakula Vinayagar Medical College and Hospital, Pondicherry, India; 5 Commissioner Office of Health and Family Welfare, Government of Telangana, Hyderabad, India; 6 Centre for Economic and Social Studies (CESS), Hyderabad, India

## Abstract

**Background:**

In India, amidst the increasing number of health programmes, there are concerns about the performance of frontline health workers (FLHW). We assessed the time utilisation and factors affecting the work of frontline health workers from South India.

**Methods:**

This is a mixed methods study using time and motion (TAM) direct observations and qualitative enquiry among frontline/community health workers. These included 43 female and 6 male multipurpose health workers (namely, auxiliary nurse midwives (ANMs) and male-MPHWs), 12 nutrition and health workers (Anganwadi workers, AWWs) and 53 incentive-based community health workers (accredited social health activists, ASHAs). We conducted the study in two phases. In the formative phase, we conducted an in-depth inductive investigation to develop observation checklists and qualitative tools. The main study involved deductive approach for TAM observations. This enabled us to observe a larger sample to capture variations across non-tribal and tribal regions and different health cadres. For the main study, we developed GPRS-enabled android-based application to precisely record time, multi-tasking and field movement. We conducted non-participatory direct observations (home to home) for consecutively 6 days for each participant. We conducted in-depth interviews with all the participants and 33 of their supervisors and relevant officials. We conducted six focus group discussions (FGDs) with ASHAs and one FGD with ANMs to validate preliminary findings. We established a mechanism for quality assurance of data collection and analysis. We analysed the data separately for each cadre and stratified for non-tribal and tribal regions.

**Results:**

On any working day, the ANMs spent median 7:04 h, male-MPHWs spent median 5:44 h and AWWs spent median 6:50 h on the job. The time spent on the job was less among the FLHWs from tribal areas as compared to those from non-tribal areas. ANMs and AWWs prioritised maternal and child health, while male-MPHWs were involved in seasonal diseases and school health. ASHAs visited homes to provide maternal health, basic curative care, and follow-up of tuberculosis patients. The results describe issues related with work planning, time management and several systemic, community-based and personnel factors affecting work of FLHWs.

**Conclusion:**

TAM study with mixed methods can help researchers as well as managers to periodically review work patterns, devise appropriate job responsibilities and improve the efficiency of health workers.

## Background

Health functionaries are integral to providing universal health coverage and attaining Sustainable Development Goals [1–4]. Frontline health workers (FLHWs) or community health workers have been at the forefront of health service delivery, especially in rural and difficult to reach settings [5–7].

A report by the World Health Organization, *A universal truth: No health without a workforce* (2013) has noted a critical shortage of healthcare staff in many countries that have reduced the range and quality of services offered [8]. Low and middle-income countries, especially, face severe challenges in ensuring a sufficient fit for purpose and fit to practice health workforce [9, 10]. Globally, two aspects have gained prominence in addressing vital issues related to human resources for health—addressing the retention of health workers and ensuring that available workers are actually at work and performing well, to provide quality health care [11].

In India, workforce shortage is manifested at all three levels of health service delivery, i.e. primary, secondary and tertiary; however, this shortage is starker in rural remote locations [12–15]. The Ministry of Health and Family Welfare, India, acknowledges that there is a shortfall of 38.2% female health assistants and 52.6% male health assistants at primary health centres (PHCs). At sub-centres (SCs), there is noted deficit of 7.8% female workers and 65.2% male workers [16]. Though the number of workers has risen significantly in recent few years, problems of imbalances in the distribution of work and continuous addition of new programs still mar health service delivery [12]. Increasingly, there is a realisation by public health managers that the FLHWs do not work properly and are underutilised, while, in contrast, the FLHWs feel that new activities are added frequently and they are burdened (experiential observation by authors). Table 1 describes FLHWs in the public rural health system in India.

**Table 1:** Frontline health workers in the public health system in India

*Ministry of Health and Family Welfare* Within the health department, PHCs are the peripheral most centres which provide the first contact between the community and a doctor in rural areas. PHCs provide basic preventive, promotive and curative, and outreach services through SCs. An SC covers a population of 3000–5000. Each SC is proposed to have one or two auxiliary nurse midwives (ANMs) and one male multipurpose health worker (male-MPHW). Previously, only one regular ANM (1st ANM) was stationed at SC, but in 2005, the Indian government introduced a contract position for 2nd ANM. At the same time, government introduced accredited social health activists (ASHAs), who are incentive-based community volunteers that serve as an interface between the community and public health system at a village level. *Ministry of Women Development and Child Welfare* The Integrated Child Development Services (ICDS) scheme aims to improve health and nutrition status of women and children. Under this scheme, nutrition centres/Anganwadi centres (AWCs) were established and services are provided by an Anganwadi worker (AWW) with support from a helper. The government envisages convergence between ANMs, ASHAs and AWWs. *For this study, the regular staff, ANMs, male-MPHW and AWWs, are referred as FLHWs while ASHAs are mentioned separately.*	

It is in this context, in the year 2015, this study was commissioned by the health department of Andhra Pradesh (before bifurcation^1^). The aim of the study was to (i) understand the role and utilisation of time by FLHWs, and factors affecting efficient utilisation of time; (ii) explore the factors that are facilitators and barriers in functioning of ASHAs; and (iii) understand the perceptions of supervisors about patterns of time spent by FLHWs and ASHAs. In this paper, we describe methods and procedures developed for the study amidst the complex nature of job activities, different geographies and interactions among FLHWs and supervisors.

## Methods

### Study area

The study was conducted in three districts from erstwhile Andhra Pradesh. Permissions for the study were obtained from state government and respective district officials. Ethics approval was obtained from the institutional ethics committee (TRC/IEC-127/2015).

### Study participants

The study comprised of two sets of participants: (i) FLHWs (ANMs, male-MPHWs, AWWs) and ASHAs and (ii) officials, such as district and sub-district public health officers, PHC medical officers and health supervisors and ICDS-project director and supervisors.

### Study design

This is a cross-sectional study using mixed methods involving a time and motion (TAM) approach [17, 18]. TAM data was collected through direct continuous observations. We conducted in-depth interviews with the participants, focus group discussion (FGD) with ASHAs and ANMs, and in-depth interviews with officials.

### Sample size and sampling

**Fig. 1:**
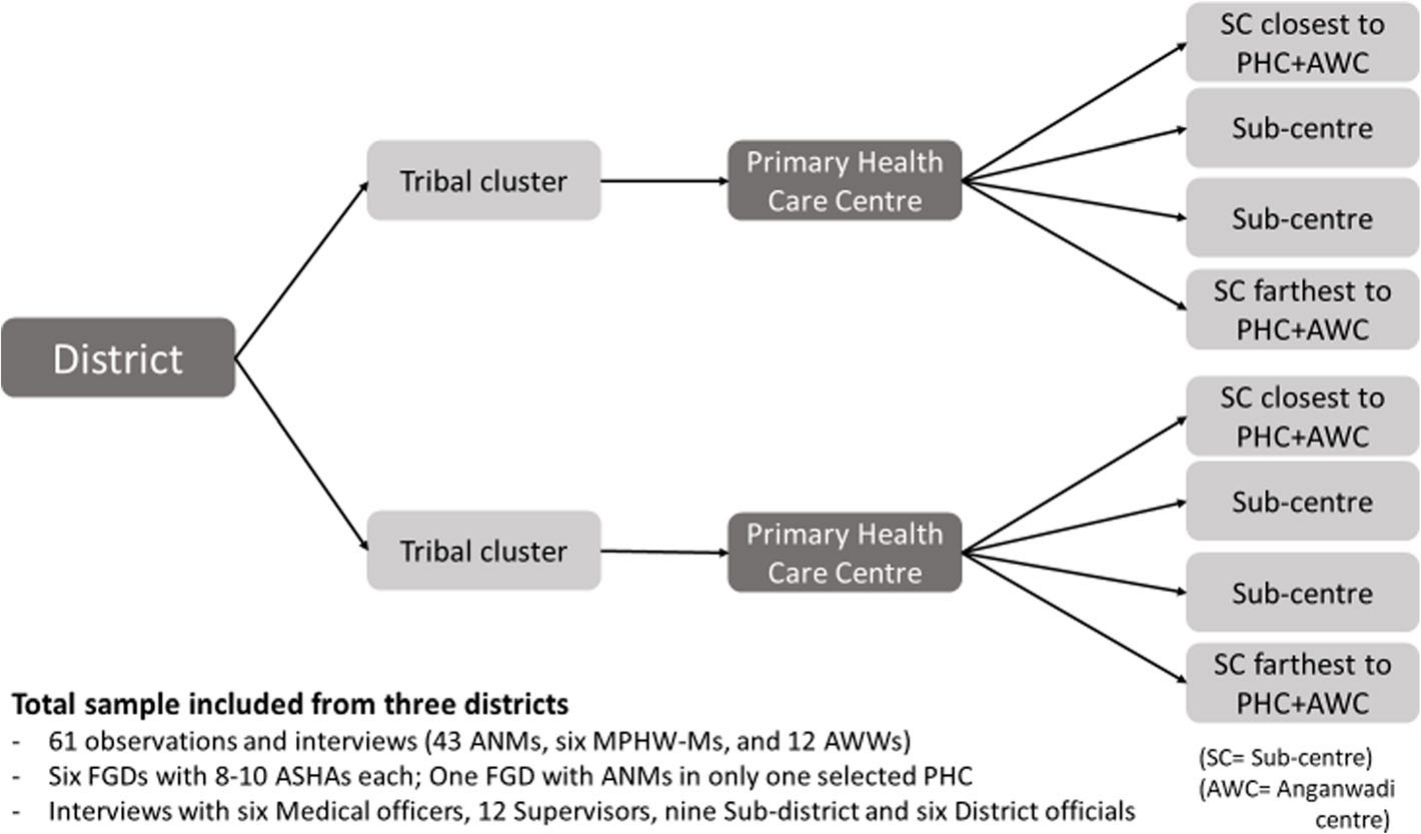
Multi-stage stratified random sampling for districts

Multi-stage stratified sampling was followed. Three districts (Srikakulum, Chittoor and Khammam) were purposively selected from three different regions of erstwhile Andhra Pradesh. Clusters (lowest administrative unit) within each district were stratified into tribal and non-tribal clusters, and one cluster was randomly selected in each stratum. Chittoor had a very small tribal population, thus we selected two non-tribal clusters. Within each cluster, one PHC was chosen at random. Under every PHC, we listed all the sub-centres which had all the desired cadre. Then four SCs were sampled based on increasing order of distance from PHC. Two AWCs, one each attached to the closest and the farthest chosen SC, were also selected. Figure 1 describes the distribution of the sampled centres. All the ANMs (43) and male-MPHWs (6) working at selected SCs and AWWs (12) working at the selected AWCs were included in the study for direct observations and interviews. For FGD with ANMs, 8–10 non-participant ANMs were sampled from sample PHC of Khammam district. All the available supervisors of the selected participants and officials at the cluster and district levels were also included in the study, total 33, for interviews.

ASHAs used their private meetings at households for most of the health education and preventive and curative services. Direct observation could be breach in the privacy of both ASHA and the visited household. Thus, direct observations were omitted; however, we conducted six FGDs with ASHAs. For FGDs with ASHAs, we sampled 8–10 ASHAs at each of the selected PHCs. We listed ASHAs in order of the lowest to highest incentive received and then sampled the desired number systematically.

The study was undertaken in two progressive phases. The first phase involved extensive formative research to understand the TAM approach (inductive vs deductive). We developed our tools based on this research and pilot tested them. In the second phase, we collected data for the main study followed by data management and analysis.

### Formative phase, August 2014–July 2015

We conducted an extensive literature review using databases such as PubMed, Medlar, Science Direct and Google Scholar to explore use of TAM in the health sector. We reviewed reports from various government ministries to understand job responsibilities and functioning of peripheral health staff, from the past 10 years. In-depth review of literature helped in identifying concerns around community-based TAM observations and developing an interview guide for interviewing experts. We then interacted with experts who conducted TAM study in Gujarat [19] and Karnataka [unpublished work of Karuna Trust and Indian Institute of Management, Bangalore]. Both studies had very small sample sizes and used inductive narrative writing for capturing activities. It emerged that they faced challenges in the management of a huge amount of collected non-structured data. Experts suggested that a deductive approach would certainly be an advantage in large sample size studies as it could result in organised and easier management of the data. We thus used an inductive approach to explore the spectrum and extent of activities performed by a small sample of FLHWs. The information thus obtained was used to develop structured formats of activity lists, with also a scope of recording ‘others’ supplemented by field notes.

#### Inductive approach—exploratory visits to SCs and AWCs

We visited two SCs and two AWCs in a non-sample district at two different times of the year. We ensured that we observed at least one immunisation day and nutrition health day. We observed 16 ANM days, six male-MPHW days and four AWW days without interfering in their routine work. On the last day, we interviewed workers in-depth to understand their job activities and work schedule-related aspects for each day of the week, as each day of the FLHW has a different schedule. We analysed this data separately for each cadre under study to identify appropriate themes and code them as a list of categories and sub-categories, and list specific activities within each category. We simultaneously explored the job responsibilities assigned by the authorities and extracted important information to complete our lists. Later, we re-visited the observed FLHWs and validated these lists, and added any missing activities. Similarly, qualitative interview schedules and FGD guides were developed by an iterative process and identifying key areas of enquiry. Tools were originally framed in English, translated into the local language and then back-translated into English.

#### Pilot testing, development of an android-based application and finalising the tools

The pilot field visits involved activities as proposed for the main study, described later. We piloted our tools twice, once by using paper-based tools and finalising the content. We faced problems in recording multi-tasking by participants. We decided to record time against the primary activity and note the secondary activity in remarks. Second pilot testing was conducted using the android-based application on tablets to check for feasibility and correctness of the application and refined it as per needs. The interview schedule and FGD guides remained paper-based. The two pilot testings were done at least 15 days apart to also capture any variation in the work schedule of workers within a month. Any new activities identified at the two times were added to the observation checklist.

### Main study August 2015–December 2015

#### Field team

We constituted a field team of 12 observers, four field supervisors and two research assistants, with relevant experience in social science research. The field team was trained through 3 days of intense classroom sessions including mock practice sessions, and 2 days of field experience in a non-selected district. Field supervisors and research assistants were also trained for conducting interviews and FGDs.

#### Data collection tools

Quantitative data on observations were collected using piloted checklists. The checklist for ANM and male-MPHW consisted of 17 major categories constituting of travel, service delivery related, and other programmatic and personal work as mentioned in Table 2. These categories were further sub-divided into sub-categories with an activity list against it. For example, under the category ‘service delivery and counselling’, one of the sub-categories devised was ‘maternal health’ with activities like registration of pregnancy, routine ante-natal check-up, referral, conducting delivery at SC and post-natal visit, counselling. For AWWs, an observation checklist had similar categories except for school health and adolescent health; however, ‘sub-categories’ and activities listed were as per their job roles.

**Table 2:** A synopsis of an observation checklist used in TAM study

S.NO	Category	Sub-category
1.	Travel	Home to field (to-fro) Home to facility (to-fro) Within field Facility to field (to-fro)
2.	Service delivery and counselling	Maternal health Child health Nutrition Communicable diseases Non-communicable diseases Seasonal diseases/epidemics/outbreaks Curative care Blindness/cataract
3.	Home visits	Health checkups Counselling
4.	Information, Education and Communication (IEC) activities among groups or at a community level	Maternal health Child health Family planning Nutrition Communicable diseases Non-communicable diseases Other health related Mobilisation Any other
5.	Health camps	No sub-category
6.	School health	IEC in schools Service delivery and counselling
7.	Adolescent health	Service delivery and counselling IEC for adolescents Any other
8.	Nutrition and health day (NHD)	IEC activity on NHD day Service delivery Any other
9.	Universal immunisation day	Administrative Service delivery Paperwork Any other
10.	Paperwork	Filling up registers Computer data entry Preparing reports Maintaining beneficiary records Any other
11.	Meetings with co-workers or village community	No sub-categories
12.	Meetings with seniors	
13.	Trainings	
14.	Administrative	
15.	Personal work	Telephonic communication Non-telephonic communication Lunch Any other
16.	Non-health- but work-related activities	Related to health department Related to other departments External agency related
17.	Waiting	For patients For staff For others
18.	Any other	

Each of the categories had further exhaustive list of activities which were to be selected based on field performance; however, time was measured only up till sub-categories

The interview schedule for FLHWs gathered information across various domains like socio-demographic and economic profile, physiological responses, information about work profile and functioning, work planning, supervision, training, and travel details. FGD guides comprised of questions on time management, supervision, facilitative factors, barriers and training needs. We used open-ended interview guides to conduct interviews with officials and explored their perception about the functioning of FLHWs.

**Fig. 2:**
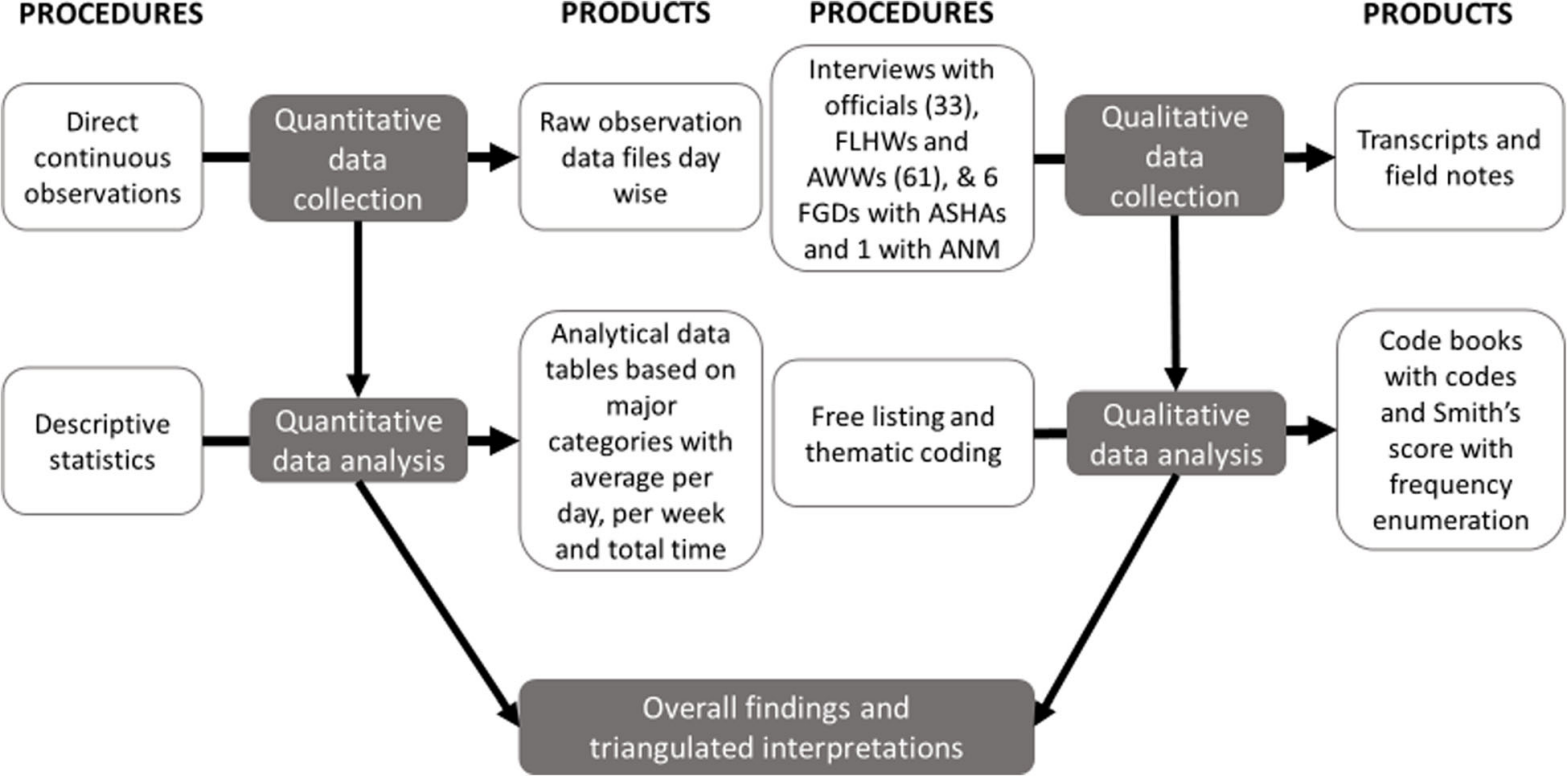
Process and products of the main study

Figure 2 below describes the processes and products of the main study.

#### Data collection process

We collected data from three districts consecutively in a span of two and half months. We observed each sampled FLHW over a span of six continuous days (Monday to Saturday) involving their daily movements from home to home. Observations and interviews were done in the natural work setting of the participants (facility or in the field). Care was taken that the observer maintained a sober profile and did not interfere in the activities of the participants. If any day of the observation week was missed, then the worker was observed on the same day in the following week by the same observer.

Interviews with FLHWs were conducted by the respective observer, in a quiet place, on the last day of observation after the working day of the health worker had ended. All FGDs with ASHAs and ANMs and interviews of supervisors were conducted by field supervisors and research assistants. Interviews with senior district officials were undertaken by the project lead. Respondent response validation was done by summarising major observations to each of the participants at the end of each interview and FGD. Wherever participants consented for audio-recording, in the end, recording was re-played for them.

ANMs who were not observed from one of the sample PHCs were included for the FGD. This FGD was conducted after preliminary analysis. The FGD was aimed towards validating the findings from preliminary analysis of data and to cover in-depth aspects related with time management, challenges, work planning, supervision, training and reporting.

#### Monitoring and supervision of data collection

In order to ensure quality data collection, stringent monitoring was devised and daily supervision was done at three levels—the first level of field supervisors, the second level of research assistants and third project lead at Hyderabad. Observation data were checked in the field at random through summary logs produced in the tablet database. The data obtained in the field was synchronised to the central server and data were uploaded on the same day of observations. This enabled to monitor data right from the beginning and track physical field movements using the inbuilt GPS mechanism in the application. Review meetings were held after data collection in each district.

#### Analysis

We conducted strengths, weaknesses, opportunities and threats (SWOT) analysis of our study methods once after finalisation of the study protocol and revised it after completion of data collection and preliminary analysis of data. We present the revised SWOT analysis in our discussion.

Quantitative data obtained from direct continuous observations were analysed for time duration per worker per day, and per week for each of the sampled PHCs and total for the district. We computed mean, range, median and inter-quartile range, and proportion of workers performing each of the categories of work. Data were analysed for each of the cadres and stratified for tribal and non-tribal clusters.

Qualitative data generated from interviews and FGDs was coded based on pre-existing themes and sub-themes. Additional data was coded under emerging themes and sub-themes. Open-ended responses were free listed, and Smith’s Saliency Score [20] value was calculated using Anthropac software. Questions asked on a Likert scale about aspects such as health status, work satisfaction, work planning, and supervision yielded data which was represented through percentages.

Overall, triangulation of information was undertaken to arrive at meaningful interpretations. The qualitative information supported and explained the reasons for the observed work pattern and time utilisation, priorities of the staff, challenges faced—systemic, community-based and personnel, and support or interference from supervisors and district administration. Preliminary findings of observations and interviews were presented to FLHWs and district officials for validation, explanations and suggestions for improving workforce efficiency.

### Ethics considerations

Written informed consent of participants was obtained before observations and interviews. Observations were non-participatory in nature disallowing any interference. Interviews were audio-recorded and photographs were taken only if respondents gave permissions. Any kind of personal identifiers was deleted during the data management process, and data were kept strictly confidential with core researchers only.

## Results

On any working day, the ANMs spent median 7:04 h, male-MPHWs spent median 5:44 h and AWWs spent median 6:50 h on the job, against expected 8 h a day. The time spent on job was less among the FLHW from tribal areas as compared to non-tribal areas. The common activities included maternal and child health, immunisation, seasonal diseases, outbreaks, home visits, school health and family planning, and education and counselling. The time was spent in other activities as well, such as adolescent health and non-communicable diseases; however, that was negligible to bring about any changes at the community level. The time spent in the stated activities varied with the type of cadre where ANMs and AWWs prioritised maternal and child health, while male-MPHWs were involved in seasonal diseases and school health. Male-MPHWs were highly underutilised by the health department. ASHAs mentioned that they visited homes to provide maternal health, i.e. follow-ups of ANC and PNC women, basic curative care to those in need, follow-ups for DOTS patient, health education and motivation.

None of the participants of any cadre had any standard work plan. There were fixed antenatal check-up, immunisation and home visit days. Apart from these, most of the work was done on an ad hoc basis. Most of the participants were not completely aware of their job responsibilities. This was more apparent in the male-MPHW cadre, though all the FLHW stated that their work was regularly disrupted by untimely last-minute meetings, trainings and other non-health related-work.

Staff stated that coordination from fellow workers, support from the community, supportive supervision and road to villages with transport facility were facilitative factors to their work, whereas non-supporting seniors, untimely disruptions due to unplanned health and non-health related work, too much documentation, rains in tribal terrain, frequent addition of new programs and activities and overburden of the various health programs reduce their work efficiency. The staff stated that new programs are added but no one helps them to assimilate these into their work plans. As a result, a new activity replaces any old activity, and activities change as the priorities of the district official’s change. A few older FLHWs also stated that their health did not support the large burden of home visits to villages which required a lot of walking by foot.

## Discussion

This manuscript presents the process for conducting community-level TAM studies in health settings. The study also focussed on qualitative aspects related to functioning and time utilisation like supervision, training, worker coordination and community participation.

The TAM approach has been successfully adopted previously to describe a typical workday of the doctor along with ways for process improvement [21, 22]. Several other studies used the TAM approach to assess work performance of different cadres of health workers [23–25] and call for standardised methods [26]. Few studies from India have also been reported from the health and nutrition sector but there are only a few studies who have directly observed community-based workers [19, 27–29]. This study is special as it has a considerable large sample size for a TAM study, considers variation across geographies, captures complex work patterns, assesses various cadres and interactions between their job activities and studies the role and perceptions of supervisors in the efficient working of FLHWs (Table 3).

**Table 3:** SWOT analysis of the study methods

Strengths	Weaknesses
• Deductive approach with a foundation of inductive approach to field inquiry. • Accurate measurement of time and better management of observation data by use of software based data collection tools. • Variations across settings (tribal, non-tribal) and cadres studied. • GPS enabled android-based app led to recording accurate movement in the field • Stringent field based monitoring and supportive supervision • A multi-disciplinary team of experts for technical guidance • Observing inter-departmental convergence	• Hawthorne effect • Inter-observer bias, due to big team of observers • Only male observers in the team, however no discomfort expressed by participants who were largely females. • Home to home follow up by a male was uncomfortable for few female workers because of social or cultural reasons • Malfunctioning of few tablets initially (two to three times in the first district).
Opportunities	Threats
• To capture best practices from the field to develop model work plan for FLHWs. • To identify policy implications and interventions for redefining job responsibilities of FLHWs. • Voicing the concerns of the lowest cadre that are unheard.	• Any punitive action against FLHWs, and ASHAs if the findings were not acceptable to higher administration. Although anonymity was maintained during reporting.

Our main strength lay in developing an extensive tool based on inductive research for recording observations and used it deductively. We left sufficient scope for incorporating any additional activities, which were outside existing list of categories and sub-categories as ‘others’ [30]. For example, category ‘non-health but work related activities’ was arrived based on worker’s own descriptions about work related with other departments, *non-government organisation*, etc. Use of android application made the task of recording and managing the observation data easier, enabled us to use GPS tracking to estimate actual distances travelled by the workers and also helped us in monitoring the field team. A SWOT (strengths, weaknesses, opportunities and threats) analysis is described in Table 3.

Although direct observations may lead to Hawthorne effect,^2^ this is likely to reduce in 2–3 days of observations. A large team would introduce inter-observer bias, which was addressed by repeat training, standardising the data collection process and on-field supervision. We also ensured that the same set of field observers were assigned the same type of health cadre. This being commissioned by the health department itself, the study has great opportunity to influence the mechanism of work planning or monitoring staff’s work efficiency. Public health managers would use the findings for better task allocation and monitoring of work. Policymakers may use the findings for re-evaluating the workload and required health workforce, to be able to provide community-level services efficiently.

## Conclusions

A TAM direct observation study complemented by extensive qualitative enquiry facilitated in assessing work patterns and time utilisation, along with issues related with work planning, time management, and several systemic, community based and personnel factors effecting work of health workers. Results of this study will generate evidence for time utilisation by workers and variation across tribal and non-tribal regions. Researchers and managers can use similar methods to periodically reviewing work patterns and devise appropriate job responsibilities to improve the efficiency of workers.

## Abbreviations

ANM: Auxillary nurse midwife
ASHA: Accredited social health activist
AWC: Anganwadi centre
AWW: Anganwadi worker
FGD: Focus group discussion
FLHW: Frontline health worker
ICDS: Integrated Child Development Services
MPHW: Multipurpose health worker
PHC: Primary healthcare centre
SC: Sub-centre
SWOT: Strengths, weaknesses, opportunities and threats
TAM: Time and motion

## Footnotes

1 The erstwhile state of Andhra Pradesh bifurcated into two states, Telangana and Andhra Pradesh, in June 2014, after the study was commissioned and protocol was developed.

2 The participants of the study may alter an aspect of their behaviour due to their awareness of being observed.
